# Creating reference gene annotation for the mouse C57BL6/J genome assembly

**DOI:** 10.1007/s00335-015-9583-x

**Published:** 2015-07-18

**Authors:** Jonathan M. Mudge, Jennifer Harrow

**Affiliations:** Wellcome Trust Sanger Institute, Cambridge, UK

## Abstract

Annotation on the reference genome of the C57BL6/J mouse has been an ongoing project ever since the draft genome was first published. Initially, the principle focus was on the identification of all protein-coding genes, although today the importance of describing long non-coding RNAs, small RNAs, and pseudogenes is recognized. Here, we describe the progress of the GENCODE mouse annotation project, which combines manual annotation from the HAVANA group with Ensembl computational annotation, alongside experimental and in silico validation pipelines from other members of the consortium. We discuss the more recent incorporation of next-generation sequencing datasets into this workflow, including the usage of mass-spectrometry data to potentially identify novel protein-coding genes. Finally, we will outline how the C57BL6/J genebuild can be used to gain insights into the variant sites that distinguish different mouse strains and species.

**Electronic supplementary material:**

The online version of this article (doi:10.1007/s00335-015-9583-x) contains supplementary material, which is available to authorized users.

## The fundamentals of gene annotation

The value of the mouse genome as a resource largely depends on the quality of the accompanying gene annotation. In this context, ‘annotation’ is defined as the process of identifying and describing gene structures. However, in the 21st century, genes are increasingly regarded as collections of distinct transcripts—generated, most obviously, by alternative splicing—that can have biologically distinct roles (Gerstein et al. 2007). The process of ‘gene’ annotation is therefore perhaps more accurately understood as that of ‘transcript’ annotation (with separate consideration being given to pseudogene annotation). The information held in such models can be divided into two categories. Firstly, the model will contain the coordinates of the transcript structure, i.e., the coordinates of exon/intron architecture and splice sites, as well as the transcript start site (TSS) and polyadenylation site (if known; see “The incorporation of next-generation sequencing technologies into mouse annotation” section). Secondly, for a transcript model to have value, it must also contain some level of ‘functional’ annotation (Mudge et al. 2013); for example, a model may contain the location of a translated region (coding sequence; CDS), alongside flanking untranslated regions (UTRs). However, our understanding of the mammalian transcriptome has evolved rapidly since the genome-sequencing era began. For example, the classical tRNA and rRNA families of small RNA (smRNA) are being joined by an ever increasing number of novel categories, including miRNAs, snoRNAs, and piwiRNAs (Morris and Mattick 2014). Of particularly interest is the discovery of thousands of long non-coding RNA (lncRNA) loci in mammalian genomes, with much of the pioneering work having being done in mouse (Carninci et al. 2005). LncRNAs—typically defined as non-coding, non-pseuodogenic transcripts larger than 200 bp—have been generally linked to the control of gene expression pathways, although a single functional paradigm seems unlikely to be established (Marques and Ponting 2014; Morris and Mattick 2014; Vance and Ponting 2014). In addition, pseudogenes—commonly described as deactivated copies of existing protein-coding genes—have long been a target for annotation projects (Frankish and Harrow 2014; Pruitt et al. 2014), and such loci can actually contribute to the transcriptome through their expression (Pei et al. 2012). Nonetheless, debate persists as to the proportion of the transcriptome that could be defined as spurious ‘noise,’ resulting from the essentially stochastic nature of transcription and splicing (Hangauer et al. 2013).

Certainly, annotation projects are under increasing pressure to provide users access to the portion of the transcriptome that is truly ‘functional’ (Mudge et al. 2013). In recent years, this process has become empowered by the advent of next-generation technologies. For example, RNAseq can be used to identify novel transcripts and to provide insights into their functionality (Wang et al. 2009), while proteomics data may allow us to finally understand the true size of mammalian proteomes (Nesvizhskii 2014). Annotation, in short, remains a work in progress, and the major challenge for the future will be to maintain the utility of the reference gene data, while providing a set of models that are an increasingly true representation of the transcriptome as it exists in nature. Here, we provide an outline of how the GENCODE project is continuing to produce comprehensive gene annotation for the reference genome of *Mus musculus*.

## Mouse GENCODE combines manual and computational annotation

**Fig. 1:**
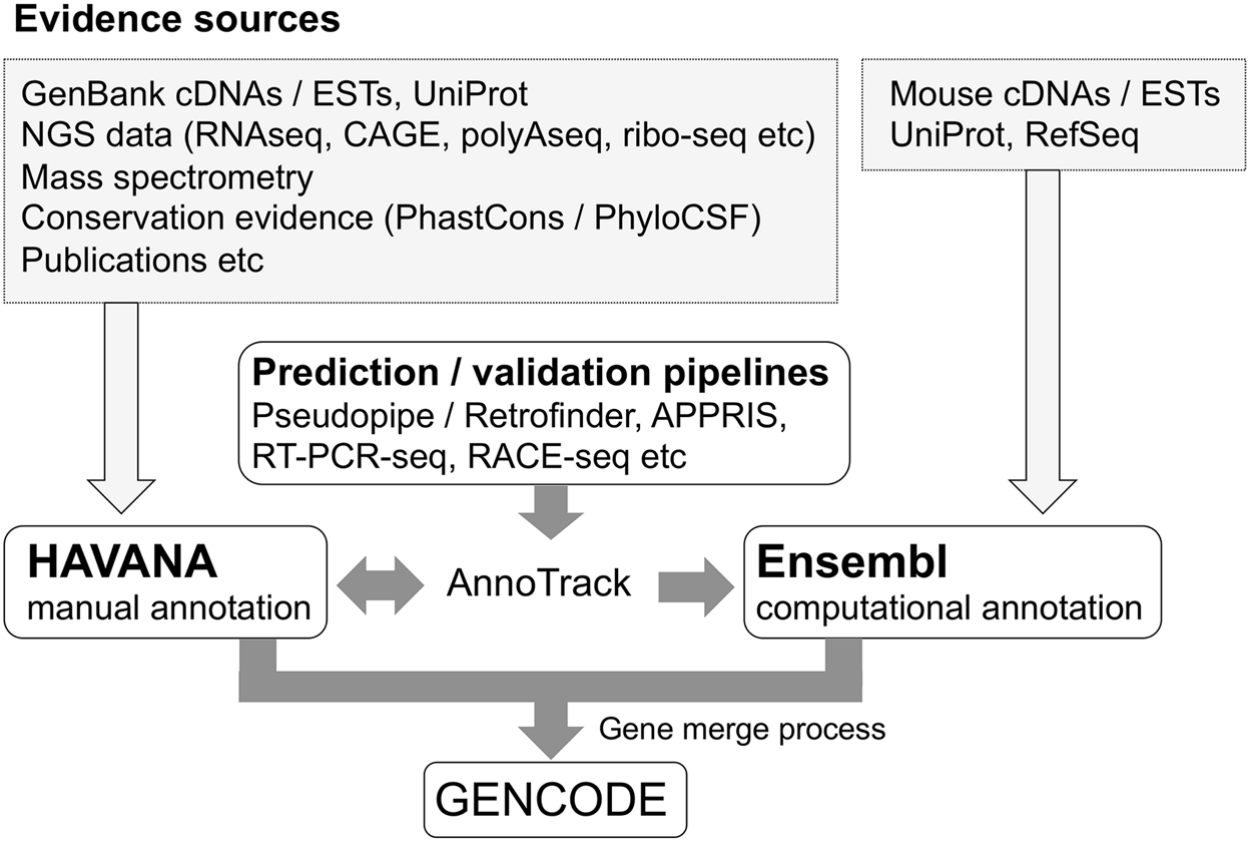
An overview of the mouse GENCODE annotation pipeline. While the HAVANA and Ensembl workflows are largely based on the alignment of Sanger-sequenced cDNAs/ESTs and protein sequences against the genome, the gene-by-gene nature of HAVANA annotation allows for further evidence sources to be incorporated. Important contributions are also made from other institutions that are part of the GENCODE project. Briefly, a subset of models are being subjected to experimental confirmation via RT-PCRseq and RACE-seq (Howald et al. 2012), in silico pseudogene models predicted using Pseudopipe (Zhang et al. 2006) and Retrofinder from UCSC are used to complement manual annotation, while the APPRIS database is used to provided inferences into the likely ‘principal variant’ of individual loci (Rodriguez et al. 2013). These contributions are monitored by HAVANA using the AnnoTrack software, which is also used to facilitate the identification and correction of putative annotation errors (Kokocinski et al. 2010)

The GENCODE project originated as an integral part of the human ENCODE project, where its remit was to identify all ‘evidence-based’ gene features found within the human genome (ENCODE Project Consortium et al. 2012; Harrow et al. 2012). More specifically, the goal of mouse GENCODE is the description of all non-redundant transcripts associated with protein-coding genes and non-coding RNAs (small and long), along with the identification of all pseudogenes. Eight institutes contribute to the project (see Acknowledgments), bringing together expertise in annotation, computational transcriptomics, experimental validation, comparative analysis, protein structure, and mass spectrometry (MS). Figure 1 summarizes the workflow of the GENCODE project. At the core of this process is manual gene annotation produced by the HAVANA group, whereby bespoke interactive software tools are used to create and appraise the alignments of a wide range of data sources—chiefly transcriptomics and proteomics data—against the genome sequence (Harrow et al. 2012; Harrow et al. 2014). To complement this work, Ensembl generates a mouse gene annotation set (henceforth ‘genebuild’) via an entirely computational process, although using similar evidence sources (Cunningham et al. 2015).

Both HAVANA and Ensembl thus build models onto the genome sequence, rather than onto transcript evidence. A disadvantage of this process is that any errors found in the genome sequence will be carried over as errors in the models. However, there are also significant reasons why genome annotation is desirable. In particular, the use of a genome scaffold for the alignment of transcriptional evidence allows for a wider variety of evidence sources to be used, including those that do not represent complete transcripts, e.g. expressed sequence tags (ESTs). Genome annotation is also better suited for the identification of pseudogenes (which may not be transcribed) (Pei et al. 2012), and can be advantageous for the interpretation of next-generation sequencing (NGS) data, as will be discussed in “The incorporation of next-generation sequencing technologies into mouse annotation” section. In fact, since HAVANA annotation is fully manual there is effectively no limit to the number of additional evidence sources that may be consulted. For example, publications based on single-locus laboratory studies often contain insights that cannot be accommodated into computational annotation pipelines, though can be effectively ‘curated’ by annotators. Critically, in-depth comparative annotation is also possible with the manual approach. This process, which essentially involves comparing the mouse genome and transcriptome against those of other species, has two major benefits. Firstly, the annotation of transcript features such as CDS can be performed (where required) with a higher degree of confidence by following the old argument that ‘conservation equals function.’ Secondly, HAVANA frequently annotates mouse models based on transcript evidence from other species—typically human or rat—when conservation is observed, thus providing additional models that seem likely to be functional.

The key stage in the creation of the GENCODE genebuild is the merging of the HAVANA and Ensembl datasets (Fig. 1). A new release is generated each time the Ensembl pipeline is re-run, approximately every three months (Harrow et al. 2012). In essence, this process merges transcripts from the two datasets that contain identical intron/exon boundaries, while maintaining models that are found in one set only. The logic behind the merge is that, while manually annotation has higher precision than computational annotation (Guigo et al. 2006), it is a much slower process. Ensembl annotation thus ‘fills in the gaps,’ covering genes and transcripts that have not yet been targeted by HAVANA. Prior to each merge process, the AnnoTrack software system is used by HAVANA to process and track both potential annotation errors and putative novel annotations suggested by the Ensembl genebuild or other GENCODE participants (Kokocinski et al. 2010). Finally, the Ensembl pipeline provides the annotation of smRNAs, based on datasets from RFAM (Griffiths-Jones et al. 2003) and miRBase (Griffiths-Jones et al. 2006). These sequences are queried against the genome with WU-BLAST, and models are constructed using the Infernal software suite (Eddy 2002).

Table 1 provides summary information on the most recent mouse GENCODE release (M5), with equivalent information provided from human GENCODE v22 for comparison. A fully comprehensive summary of the mouse release is presented in Supplementary Table 1. While the HAVANA group has performed manual annotation on the entirety of the human reference genome, the same is not true for the mouse genome. As such, the proportion of mouse GENCODE models that are computationally derived is higher than for the human genebuild. For example, 1 % of protein-coding genes and 11 % of protein-coding transcripts in human are Ensembl-only, compared with 26 % and 20 % in mouse, respectively. It is this factor—rather than underlying biological differences between the two species—that is likely to explain the more obvious tallying divergences. Firstly, the HAVANA group has thus far allocated more resources to the annotation of lncRNAs and pseudogenes in human than in mouse (Derrien et al. 2012; Pei et al. 2012). Conversely, mouse GENCODE currently has over 2000 more protein-coding genes than human. While this observation may actually have at least a partial biological explanation—most obviously, the mouse olfactory gene family is substantially larger than in human (Niimura and Nei 2005)—we anticipate that a significant part of this excess represents either spurious computational CDS predictions on lncRNAs or loci that will be re-annotated as pseudogenes in due course.

**Table 1:** A summary of mouse GENCODE annotation release 5, compared against human 22

Images have been collated from the GENCODE webportal (www.gencodegenes.org), which is immediately updated for each new genebuild release. Only major annotation categories or summary counts are shown here; for more detailed counts—e.g., relating to individual long non-coding RNA loci, transcribed versus non-transcribed pseudogenes, immunoglobulin/T cell receptor loci, etc—please consult Supplementary Table 1 or the webportal. Note that the difference in the number of protein-coding transcripts versus the total number of distinct translations within each genebuild is due to the existence of identical CDS on multiple models, typically resulting from alternative splicing within untranslated regions

Thus far, the HAVANA group has approached mouse annotation from a variety of directions. Initially, four chromosomes sequenced at the Wellcome Trust Sanger Institute (2, 4, 11, and X) were systematically annotated on a clone-by-clone basis during the assembly phase. Secondly, numerous genomic regions and gene families considered of particular interest to the wider community had their annotation prioritized, for example, the major histocompatibility complex on chr17 (unpublished), the Major Urinary Proteins gene cluster on chr 4 (Mudge et al. 2008) and the large complement of immunoglobulin loci found at several sites across the genome (unpublished). The HAVANA group has also been involved in several collaborative projects over the years that have required annotation on a gene-by-gene basis. Examples include the consensus CDS project (CCDS) (Farrell et al. 2014), which produces a set of CDS that are agreed upon by HAVANA, Ensembl and RefSeq, and the European Conditional Knockout Mouse Consortium (EUCOMM), in which 1000 mouse protein-coding genes were annotated as part of the wider International Knockout Mouse Consortium (IKMC) to aid phenotype-based investigations into their function (Bradley et al. 2012). Currently, the HAVANA group is funded by the GENCODE consortium to resume systematic chromosome annotation. Efforts are largely focused on loci not already covered by the EUCOMM/IKMC or CCDS work, which are typically lncRNAs and pseudogenes. However, improvements to protein-coding genes are being made as required.

**Fig. 2:**
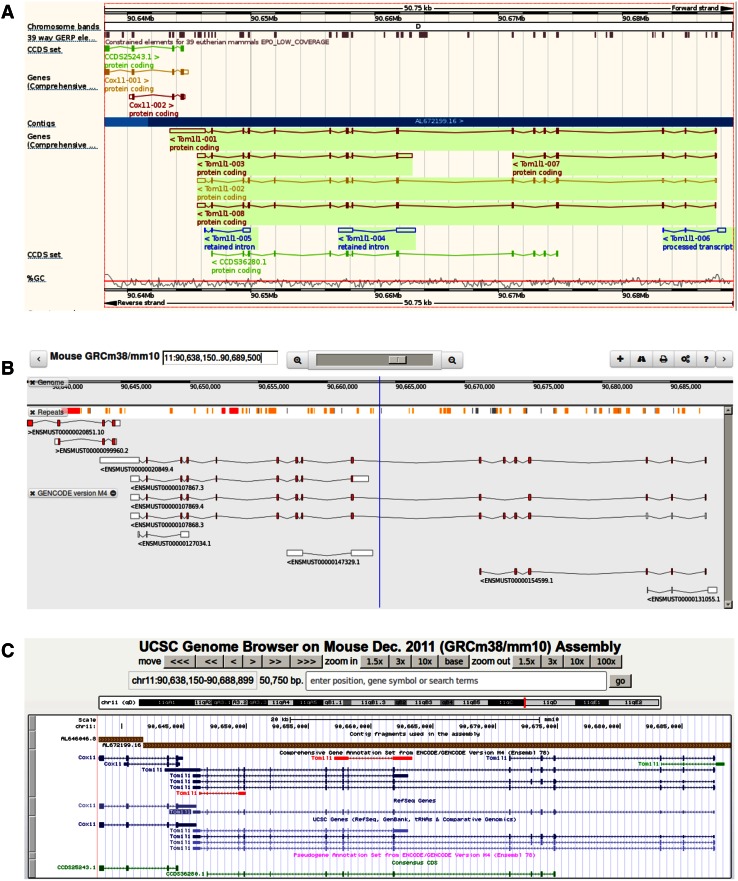
Viewing mouse GENCODE annotation in the Ensembl, Biodalliance, and UCSC genome browsers. Mouse GENCODE is the default annotation in the Ensembl genome browser (**a**), while the GENCODE webportal contains an embedment of the Biodalliance genome visualization tool (**b**). Both browsers will always feature the most up-to-date genebuild. GENCODE can also be viewed in the UCSC genome browser (**c**; the ‘UCSC Genes’ and ‘RefSeq Genes’ annotation tracks are shown for comparison), although a new release does not become immediately available; version M4 is displayed here. In each screenshot, the ‘Comprehensive’ GENCODE annotation is presented for the adjacent genes *Cox11* and *Tom1l1*, thus showing all GENCODE models associated with these loci. The Ensembl and UCSC browser screenshots also display the Consensus Coding Sequence (CCDS) project models for these loci (Farrell et al. 2014), colored *green* in both cases

GENCODE annotation is presented as default in the Ensembl genome browser (see Fig. 2a), In addition, the GENCODE webportal (www.genecodegenes.org) features an embedment of the Biodalliance genome visualization tool (Fig. 2b), allowing users to create their own integrated view of GENCODE transcript models alongside their own experimental datasets (Down et al. 2011). GENCODE can also be viewed in the UCSC genome browser (Rosenbloom et al. 2015) (Fig. 2c). In contrast, HAVANA manual annotation alone is represented in the specially designated VEGA genome browser (Harrow et al. 2014). Since this annotation is continually updated, VEGA provides users with access to the most up-to-date HAVANA models prior to the each GENCODE release. The genebuild itself can be obtained from the GENCODE webportal or from the Ensembl site (Cunningham et al. 2015). The GENCODE genebuild is currently available as a GTF file in two forms, ‘Comprehensive’ and ‘Basic’ (Harrow et al. 2012). While Comprehensive includes all GENCODE annotation, Basic contains only full-length coding transcripts (i.e., where initiation and termination codons are found), and transcripts annotated with one of the subcategories of lncRNA. One of the major advantages of the GENCODE genebuild is that it contains a sophisticated system of gene-level and transcript-level classifications—termed ‘biotypes’—as summarized in Supplementary Table 1. Essentially, gene-level classification separates protein-coding genes, lncRNAs, and pseudogenes, while the wider variety of transcript biotypes provides inferences into the functionality of individual models (Harrow et al. 2012). Of particular note, GENCODE (unlike other genebuilds) describes transcripts likely to be targeted for degradation by the nonsense-mediated decay surveillance pathway (NMD) (Mendell et al. 2004). NMD models are manually annotated, and contain the CDS predicted to trigger this process. GENCODE biotypes can be also used to filter the genebuild; for example, a user may wish to discard all transcripts associated with protein-coding genes that are not themselves annotated as protein-coding. GENCODE also contains a number of fixed-vocabulary ‘attributes’ attached to particular genes or transcript models within the GTF file. Attributes fall into three categories—pertaining to splicing, translation, or transcriptional support—and provide additional insights into the annotation of a gene or transcript model. For a full list of GENCODE attributes, consult the release notes provided at the GENCODE webportal. Finally, the GTF file also reports whether a model has been created by the manual annotation process or is instead a computational prediction. Note that when HAVANA and Ensembl biotypes conflict during the merge of particular models, the HAVANA decision is given priority.

## The incorporation of next-generation sequencing technologies into mouse annotation

HAVANA and Ensembl annotation efforts on the mouse draft genome sequence began in 2000. For most of this first decade, annotation was almost entirely based on Sanger-sequenced transcriptomics data, i.e., all publically available cDNAs/mRNAs and ESTs. In more recent years, next-generation technologies have transformed RNA sequencing (Robertson et al. 2010), and these datasets offer the potential to similarly transform the annotation process. Nonetheless, the nature of these datasets provides challenges for such endeavors, largely because (1) the amount of data produced in a typical NGS experiment is enormous, and (2) NGS reads (especially those produced by the first wave of sequencing platforms) are typically far shorter than the RNAs from which they are captured, complicating efforts to map these reads to the genome and to generate full-length transcript models (Engstrom et al. 2013; Steijger et al. 2013). It would be fair to say that the computational difficulties inherent in NGS data analysis continue to place limitations on the incorporation of these resources into annotation projects. Nonetheless, mouse GENCODE currently benefits from the inclusion of NGS data from a variety of sources. Most obviously, RNAseq can provide the core evidence of transcribed regions, including splice junctions, while CAGE (Cap Analysis of Gene Expression) and polyadenylation sequencing (polyAseq) allow for the transcription start and end points, respectively, to be confirmed. The CAGE protocol specifically targets the 5′ capped region of RNA molecules, generating large datasets of short sequence tags that can be mapped to the genome and used to infer the locations of transcription start sites (TSS) (Shiraki et al. 2003; Takahashi et al. 2012). In particular, the FANTOM consortium has generated extensive, tissue-specific mouse CAGE libraries as part of the FANTOM5 project (Forrest et al. 2014) (see also de Hoon et al. in this issue). Analogously, the polyAseq protocol as used by Derti et al. targets the site of RNA molecules where the polyadenylation tail is added to the maturing transcript (Derti et al. 2012). As for CAGE, large numbers of short sequence reads are mapped onto the genome and extrapolated into polyadenylation sites.

**Fig. 3:**
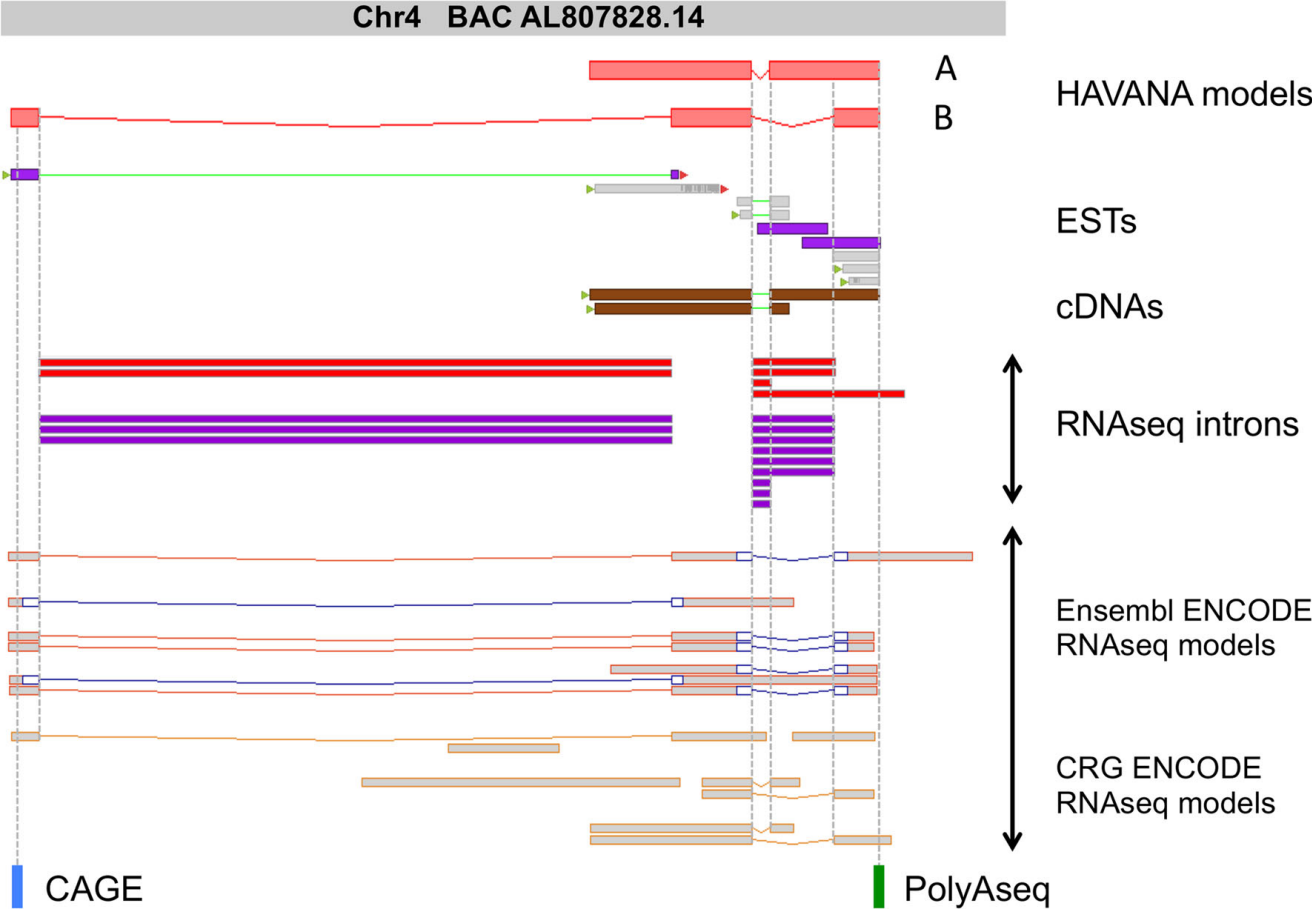
The NGS-supported annotation of a novel mouse lncRNA locus. Two HAVANA-annotated lncRNAs transcripts (*A* OTTMUST00000139812; *B* OTTMUST00000020448) found within the same gene (OTTMUSG00000009012) are displayed. Model A was annotated initially, based on Sanger-sequenced transcriptomics data; model B was subsequently added based on the NGS data. These supporting evidence sets are displayed below as follows, from *top* to *bottom*: mouse ESTs; mouse cDNAs; introns supported by Illumina RNAseq data (i.e., split reads) obtained separately from David Adams at WTSI (*red*; ArrayExpress ID: E-MTAB-599) and ENCODE (*purple*), both processed by the Ensembl RNAseq pipeline; RNAseq models based on ENCODE data, separately constructed using the Ensembl and CRG RNAseq pipelines; polyAseq site and filtered CAGE transcription start site regions predicted by Derti et al. (Gene Expression Omnibus ID: GSE30198) and FANTOM (DDBJ accession: DRA000991), respectively. The presence of CAGE and polyAseq data at the start and end point of transcript B confirms that the complete model has been annotated

**Fig. 4:**
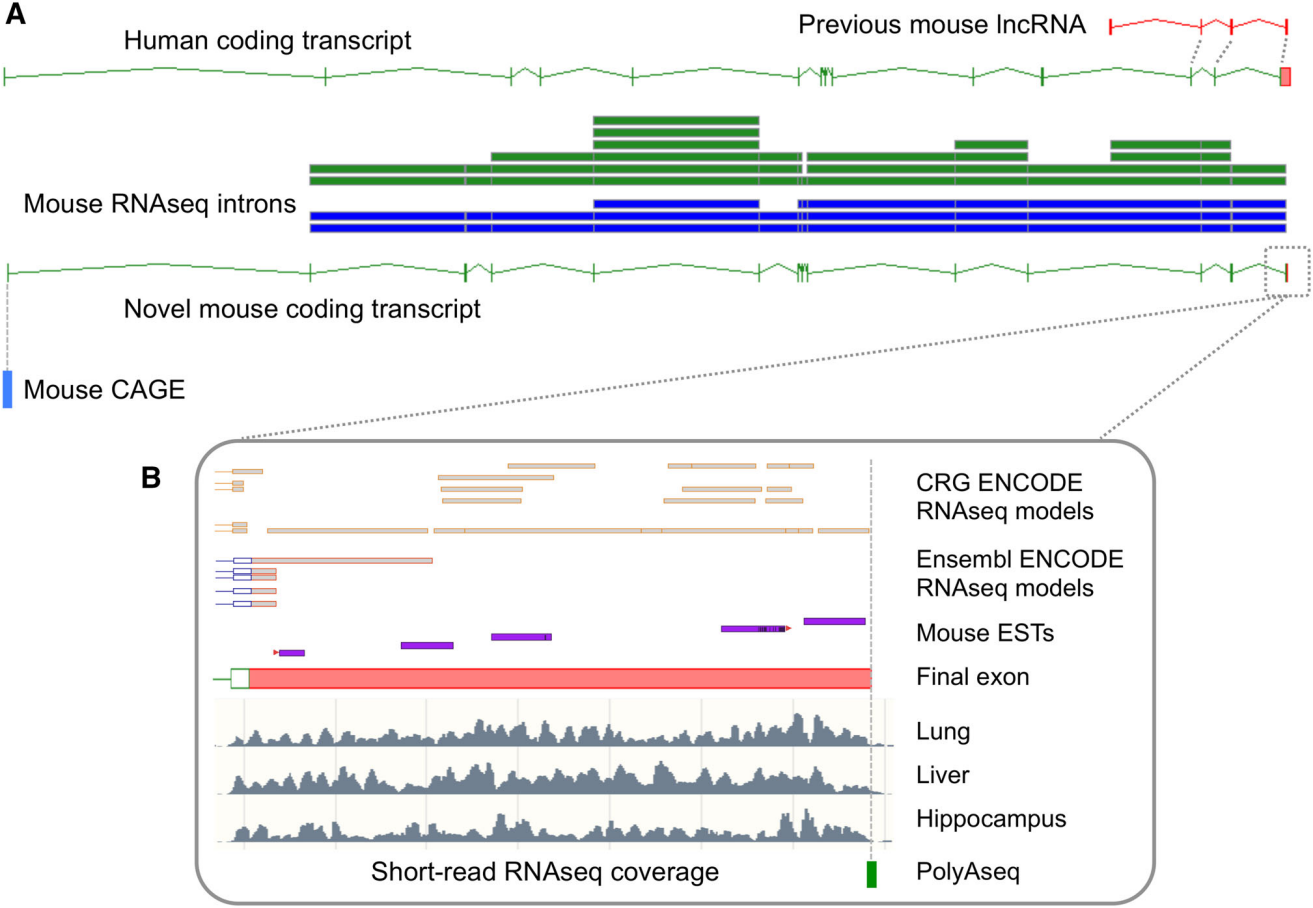
The re-annotation of a protein-coding gene in mouse GENCODE. **a** Originally, a single mouse lncRNA transcript (OTTMUS00000129569; *top* of diagram) was annotated based on cDNA AK012899.1 (not shown), creating gene OTTMUS00000051138. During reappraisal, comparative annotation and RNAseq analysis showed that the locus is orthologous to human protein-coding gene *NAALADL2* (major transcript OTTHUMT00000347390 is shown), allowing for the generation of novel mouse protein-coding transcript OTTMUS00000140064, and the reclassification of OTTMUS00000129569 as a non-coding transcript. Split read-supported introns are shown in *blue* and *green*, from David Adams at WTSI (ArrayExpress ID: E-MTAB-599) and ENCODE, respectively, both processed by Ensembl. CAGE data from FANTOM5 (DDBJ accession: DRA000991) support the presence of a TSS for OTTMUS00000140064. **b** While ESTs and RNAseq models did not allow for the final exon to be annotated with confidence, its structure could be resolved manually based on RNAseq read coverage graphs (three examples from David Adams data are shown) alongside polyAseq data from Derti et al. (Gene Expression Omnibus ID: GSE30198). Several non-coding EST-based transcripts subsequently added as models at the 5′ end of the locus are not featured. The locus spans approximately 1 Mb of genomic sequence

From the outset, NGS sequencing data can benefit genebuilds both by identifying new genes and transcripts and by allowing for improvements to be made to existing models. Such improvements may involve the completion of partial models, although NGS datasets can also provide significant insights into how (or indeed if) transcripts actually function. These inferences can then be passed onto users through the GENCODE functional annotation system, as described in “Mouse GENCODE combines manual and computational annotation” section. The novel genes being added to mouse GENCODE as part of this work are almost entirely lncRNAs. Figure 3 illustrates the annotation of one such locus. In this example, RNAseq data processed by Ensembl and/or the Centre de Regulació Genòmica (CRG) highlighted potential novel introns within a HAVANA lncRNA, and this locus was subjected to manual re-annotation. Transcript A alone had been created initially, based on two cDNAs, while transcript B was annotated later based on the NGS datasets. The two introns of transcript B are supported by two RNAseq studies, although since these experiments used short RNAseq reads, a further level of manual interpretation was required to extrapolate complete transcript structures. Transcript B was considered a reasonable extrapolation because in one RNAseq dataset set the two introns are found in spleen and thymus tissues. In contrast, the longer second intron found in the same set was not converted into annotation as it is only found weakly expressed in a single tissue and is not recapitulated in the ENCODE data. The manual process also allowed the lack of coding potential to be reappraised—the putative ORFs suggested in certain of the RNAseq models were rejected as spurious—and the gene was ‘biotyped’ as an antisense lncRNA. In contrast, Fig. 4 details the re-annotation of a mouse lncRNA into protein-coding gene *Naaladl2*. In this example, a large protein-coding gene was not originally apparent due to a lack of cDNAs or ESTs in the region. In fact, a short, single-transcript gene was initially annotated in the region, biotyped as a non-coding model because a plausible CDS could not be identified. When reappraised, a comparative analysis made it clear that this transcript was partially orthologous to human protein-coding gene *NAALADL2*, and that each of these additional human introns has support in mouse RNAseq libraries. This allowed for the construction of a conserved 795aa protein-coding transcript in mouse, while the original lncRNA transcript could now be seen to be a non-coding alternative transcript. In addition, it proved possible to extrapolate the true extent of the final exon by manually appraising RNAseq read coverage graphs alongside polyAseq data (Fig. 4b).

As noted in “Mouse GENCODE combines manual and computational annotation” section, GENCODE contains a large number of incomplete models, and RNAseq data can now be used to ‘complete’ these. While the presence of partial models in GENCODE allows users to work with exons and splice junctions that may nonetheless be biologically important, one issue is that the functional annotation of such models tends to be more predictive. In fact, even when a model is based on a cDNA, it cannot be assumed that the sequence captured is full-length, i.e., contains the true TSS or endpoint. However, the observation of significant CAGE data at the beginning of other transcript evidence can be used to confirm that the TSS has been found, adding confidence to the subsequent functional annotation. For example, in Fig. 3, the presence of CAGE data at the start of model B indicates that exons are not missing at the 5′ end, ruling out the possibility that a 5′ extension to the model could uncover a legitimate CDS. Transcript endpoints can be identified with polyAseq tags, and these datasets actually suggest that the 3′ UTRs of human and mouse models in GENCODE are frequently too short. In fact, polyAseq data and regular RNAseq are readily combined during manual annotation to resolve the true extent of 3′ UTR sequences (Fig. 4b). Furthermore, using such data, HAVANA has been able to identify and reclassify dozens of transcripts that were incorrectly classified as lncRNAs when in fact they represented extended 3′ UTRs of upstream protein-coding genes (unpublished observation). Finally, note that HAVANA annotates polyadenylation features (both sites and regulatory signals) directly onto the genome sequence, and that these features are not explicitly linked to individual transcript models. Similarly, at the present time, HAVANA does not annotate additional transcript models where the only difference is in the usage of distinct polyadenylation features.

While RNA sequencing methodologies are providing clear insights into the size of the transcriptome, the size of the proteome remains far harder to elucidate. For the most part this is because, while alternative splicing has the potential to generate large numbers of alternative protein-isoforms, a minority of alternative transcripts have had their functionality experimentally confirmed (Mudge et al. 2013). As such, a significant amount of the CDS annotation in GENCODE is considered ‘putative.’ The underlying problem is that it is far harder to obtain protein sequences than it is to obtain RNA or DNA sequences (Faulkner et al. 2015). However, from an annotation perspective at least, the situation is improving. Firstly, ribosome profiling (RP; also known as Ribo-seq or ribosome footprinting) provides a way around the difficulties in dealing with protein molecules by instead capturing and sequencing fragments of RNA that are bound to ribosomes (Ingolia et al. 2009; Ingolia et al. 2011; Lee et al. 2012). This technique can be modified to specifically map initiation codons, with obvious potential benefits to annotation pipelines. Nonetheless, it should be emphasized that RP maps sites of ribosome occupancy on RNA molecules; it does not obtain actual protein sequences, and debate about the correct way to interpret these data is ongoing (Ingolia 2014). At the present time, HAVANA only uses RP data to resolve situations where it is not obvious which initiation codon to use in a CDS.

Secondly, advances in MS have led to a significant increase in the number and quality of deduced peptide sequences becoming available to annotation projects (Yates 2013; Nesvizhskii 2014), leading to a similar expansion in the number of repositories to hold such data (Perez-Riverol et al. 2014). While MS peptides can be used to validate existing CDS, the greater interest for annotation projects at the present time is in the discovery of novel CDS. In fact, a pair of recent publications claimed that there may be significant numbers of missing protein-coding genes in the human genome, based on MS-supported novel translations found in transcribed regions out with the current set of protein-coding genes (Kim et al. 2014; Wilhelm et al. 2014). However, the validity of these interpretations has been called into question (Ezkurdia et al. 2014). We believe that both the calling of peptide-spectrum matches (PSMs) and the mapping of these sequences back to the genome should be based on highly conservative parameters (Brosch et al. 2011). Furthermore, the interpretation of PSM to genome alignments should be subjected to manual scrutiny. In this way, we observe that PSMs that do not fall within known protein-coding genes are commonly associated with pseudogenes. Furthermore, PSMs within pseudogenes or lncRNAs frequently cannot be linked to canonical initiation codons upstream (unpublished observations). Essentially, HAVANA does not make protein-coding genes solely based on MS data where either the evidence is equivocal or the biological interpretation is unclear. As a consequence, neither the mouse nor the human GENCODE genebuilds currently contain ‘orphan’ proteins—i.e., CDS that lacks orthologs or paralogs in other species—where the only supporting evidence for translation is PSMs from MS experiments. However, orphan proteins could theoretically be added to these genebuilds in the future, provided this annotation is supported by vigorous functionality based experimental studies.

## New horizons—the annotation of other mouse strains

To date, mouse GENCODE annotation has focused on the reference genome of *Mus musculus*, strain C57BL/6J (Waterston et al. 2002). However, a major interest in mouse genomics is to identify differences both between distinct mouse species and laboratory strains of the same species. Over the last decade, the HAVANA group has worked on a number of alternative mouse genomes as part of external collaborations. For example, candidate Insulin-dependent diabetes (*Idd*) regions on six chromosomes have also been annotated in one or more of the NOD/MrkTac, NOD/ShiLtJ, and 129 strains (Steward et al. 2013). Today, researchers have increasing access not just to regions of alternative mouse genomes, but to the entire genomes themselves (Yalcin et al. 2012). In particular, the Mouse Genomes Project is an ongoing effort to provide high-quality genome sequences for both classical laboratory strains and wild-derived inbred mice; see Adams et al. in this issue. While variant sites can be imputed from such alternative genomes and simply displayed against the reference mouse genome [for example, using the BioDalliance tool at the GENCODE webportal (Down et al. 2011)], the interpretation of such variation is made easier if alternative annotation models are also available. This is especially true when considering structural variation, which has been a focus of comparisons between mouse genomes (Yalcin et al. 2011; Keane et al. 2014). Annotation projects are particularly interested in large-scale structural variation, as this phenomenon is often linked to changes in gene copy number; such events may be of interest to both medical and evolutionary biologists (Bailey and Eichler 2006; Chain and Feulner 2014). In our experience, manual annotation is highly desirable for such complex regions; computational analysis pipelines may fail to interpret the correct evidence for a particular gene copy, especially where several genes have highly similar sequences, and may also fail to correctly identify pseudogenization events.

For the last few years, the mouse reference assembly has been improved under the guidance of the Genome Reference Consortium (GRC) (Church et al. 2011). The first remit of the GRC is to fix errors and close sequence gaps in the draft sequence. In the former case, the HAVANA and RefSeq groups play a key role in identifying indels and nonsense mutations within mouse protein-coding genes. These findings are reported to the GRC, and when the sequence region has been reappraised the results are fed-back to curators, who update the gene annotation if necessary. For example, a protein-coding gene with a putative sequencing error may turn out to be a genuine pseudogene. The GRC also provides alternative assemblies (‘alt loci’) of regions that are variable between genomes (http://www.ncbi.nlm.nih.gov/projects/genome/assembly/grc/). The *Idd* regions annotated by HAVANA are now included in the GRC as alt loci. In total, GRCm38.p3—the version of the mouse reference genome released in March 2014—contains 99 alt loci, featuring sequence from 13 additional mouse genomes. All alt loci produced by the GRC will be incorporated into the GENCODE genebuild. In due course, the complete genome sequences provided by the Keane group will be added to the mouse GRC repository, and they will become targets for manual annotation. It is both unfeasible and unnecessary that each of these genomes will be subjected to complete manual annotation. We anticipate that a large proportion of the existing reference assembly annotation models will simply be ‘lifted across’ between genomes. Manual annotation will then be employed to (a) investigate and improve loci that have failed to project successfully, and (b) to specifically target regions of known genomic complexity—e.g., dynamically evolving gene families—where accurate annotation is likely to be particularly difficult. Furthermore, the manual annotation process will once again provide an important ‘QC’ service on these sequences, helping to distinguish true variant sites from artifacts or errors that arose during the genome sequencing, assembly, or alignment stages.

## Future prospects

The GENCODE annotation of the mouse reference genome is continuing along several fronts. Firstly, not all gene features are represented at the present time, in terms of exons, transcripts, and even whole loci (Mudge et al. 2013; Cunningham et al. 2015). The mouse GENCODE gene and transcript counts are thus expected to rise consistently over the coming years as manual annotation continues and further transcript libraries become available. However, while the number of RNAseq reads available already runs into the hundreds of millions, concerns have been raised about the power of this technique to find transcripts with very low expression levels (Oshlack and Wakefield 2009). CaptureSeq is proving to be highly useful in this regard, being a method by which transcripts with extremely low expression can be enriched through the use of tiling arrays designed across regions of interest (e.g., intragenic space) prior to high-depth sequencing (Mercer et al. 2012; Clark et al. 2015). We anticipate that this methodology will be used to uncover new mouse lncRNAs, in particular those with restricted expression profiles.

Secondly, a significant amount of work remains to be done in the functional annotation of the mouse transcriptome, in particular in allowing users to distinguish transcripts that are biologically interesting from those that are not. While the completion of mouse (or human) functional annotation cannot be considered a short-term goal, we anticipate that annotation projects such as GENCODE will be able to make significant progress over the next few years. Initially, the completion of currently incomplete GENCODE models will be of enormous assistance in this regard. Here, we have outlined methodologies for model completion that can be carried out at the present time based on short-read RNAseq coverage graphs and models, as well as CAGE and polyAseq. However, longer RNAseq read libraries are becoming available using platforms such as PacBio (these data are already proving useful for human annotation (Sharon et al. 2013))—while nanopore-based RNA sequencing is on the horizon (Clarke et al. 2009)—and in due course we anticipate that true full-length RNA sequences will negate the need to combine RNAseq with separate end-sequencing protocols (Picelli et al. 2014).

 Another advantage of NGS is that insights can be gained into levels of transcription, which can be compared—for example—between tissues or developmental stages (Wang et al. 2008; Lin et al. 2014). For the human transcriptome, several projects have already sought to identify ‘dominant’ transcripts; i.e. the transcript (or protein) in a particular gene that has the highest, most consistent level of expression (Djebali et al. 2012; Gonzalez-Porta et al. 2013; Ezkurdia et al. 2015). In the near future, improvements to RNAseq technologies will complement the maturation of single-cell protocols, allowing us to observe changes in transcript expression profiles with increasing accuracy and resolution. Meanwhile, functional transcripts can also be extrapolated based on their evolutionary conservation (Fig. 4a). GENCODE is integrating the output of the APPRIS pipeline, which aims to identify the ‘principal’ RNA produced by a gene on the basis of exonic conservation (alongside inferences made into the protein structure) (Rodriguez et al. 2013). For mouse and human GENCODE, the principal APPRIS isoform for each protein-coding gene is designated in the GTF file, or if no model matches these strict criteria a single ‘candidate’ model can instead be selected based on its score or length. We emphasize that such methodologies extrapolate functionality through the use of proxies, and that the true descriptions of functionality must ultimately come from single-gene laboratory studies. Even so, we would argue strongly that annotation projects such as mouse GENCODE must do all they can to provide guidance into transcript functionality at the present time, given that the high demand for this information. For example, the development of the CRISPR/Cas system for genome engineering is completely changing the landscape of mouse genomics, offering a simple method by which mouse genes can be disrupted or switched on and off (Jinek et al. 2012; Mali et al. 2013; Qi et al. 2013; Wang et al. 2013). However, uncertainties regarding the functionality of transcriptional complexity within genes, antisense to genes, and within intragenic space currently represent hurdles to both the design of CRISPR/Cas assays and the interpretation of the results produced. In a wider context, gene annotation will always be an integral component of genome science, from medical to evolutionary biology. It is therefore important that all steps are taken to ensure that genebuilds are as accurate and comprehensive as possible.

## Electronic supplementary material

Supplementary Table 1: An extended description of mouse GENCODE annotation release 5. This table provides the full list of GENCODE gene and transcript biotypes used to produce the summary for release M5 presented in Table 1. This is a screenshot obtained from www.genecodegenes.org/mouse_stats/current.html. Supplementary material 1 (TIFF 13928 kb)
