# Supplementary figures and images for: Creating reference gene annotation for the mouse C57BL6/J genome assembly
Source: Mamm Genome. 2015 Jul 18;26(9-10):366–78. doi: 10.1007/s00335-015-9583-x (PMC4602055; doi:10.1007/s00335-015-9583-x)

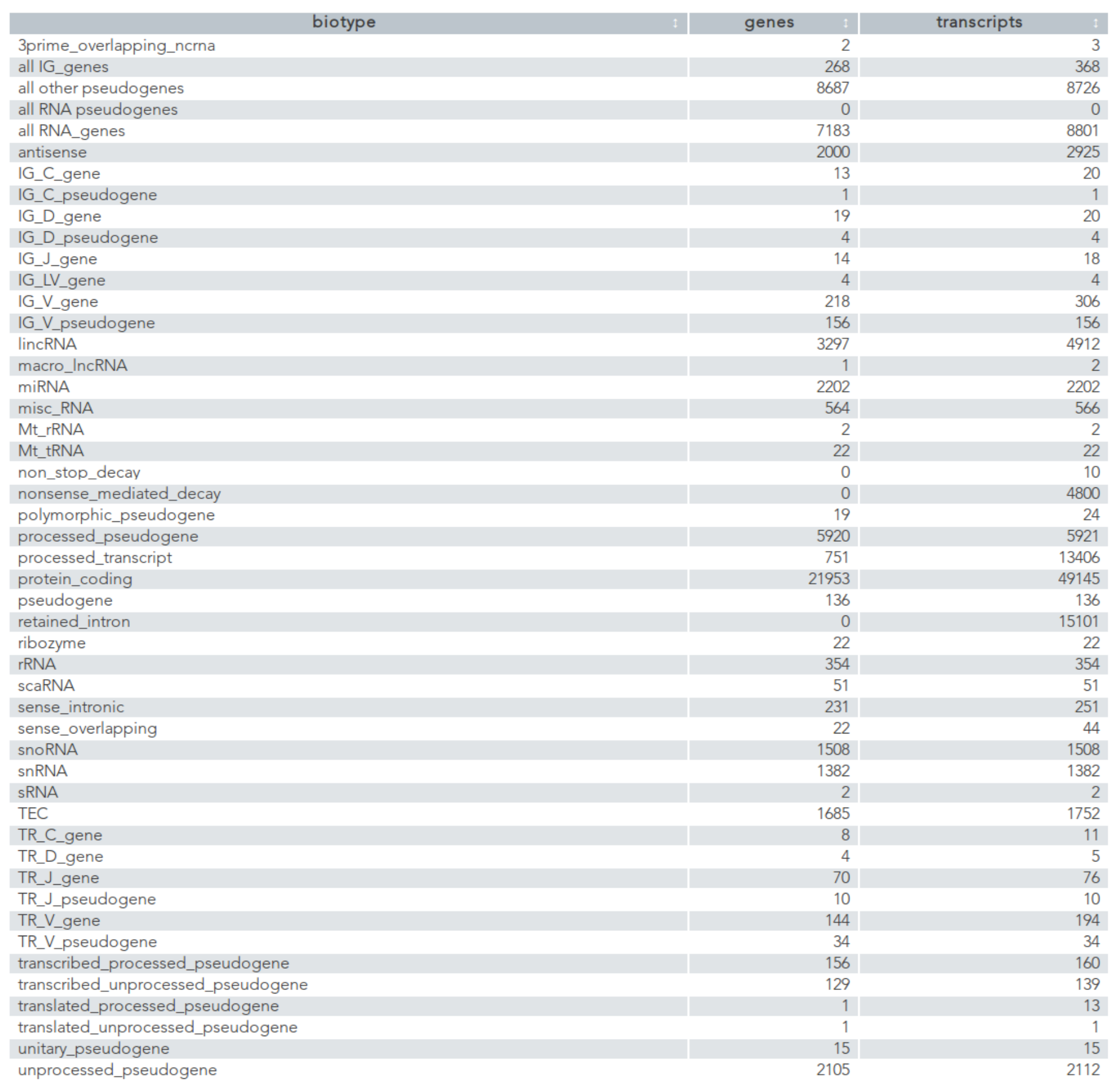

Supplement: Supplementary file 1 — Supplementary Table 1: An extended description of mouse GENCODE annotation release 5. This table provides the full list of GENCODE gene and transcript biotypes used to produce the summary for release M5 presented in Table 1. This is a screenshot obtained from www.genecodegenes.org/mouse_stats/current.html. Supplementary material 1 (TIFF 13928 kb) [file 335_2015_9583_MOESM1_ESM.tiff]
